# Supplementary material for: Cellular Changes in Injured Rat Spinal Cord Following Electrical Brainstem Stimulation
Source: Brain Sci. 2019 May 28;9(6):124. doi: 10.3390/brainsci9060124 (PMC6628227; doi:10.3390/brainsci9060124)
Supplement: Supplementary file 1 [file brainsci-09-00124-s001.pdf]

Supplementary Materials: Cellular Changes in Injured Rat Spinal Cord Following Electrical Brainstem Stimulation

Table S1. List of antibodies and concentrations used.

Antibody	Manufacturer	Host	Dilution
Primary			
**BrdU**	Novus	Rat	1:100
**BrdU**	Roche	Mouse	1:100
**NeuN**	Millipore	Mouse	1:1000
**Blbp**	Abcam	Rabbit	1:100
**Sox2**	Santa Cruz	Rabbit	1:500
**GFAP**	Invitrogen	Rat	1:500
**CD68**	Millipore	Mouse	1:500
**Double cortin**	Millipore	Guinea Pig	1:100
**APC**	Abcam	Mouse	1:500
**Arginase-1**	Invitrogen	Rat	1:200
**iNOS**	Novus	Rabbit	1:500
**NG2**	Millipore	Mouse	1:500
Secondary *(488, 594, 647 nm)*			
**Mouse**	Alexa Fluor	Goat	1:10,000
**Rabbit**	Alexa Fluor	Goat	1:10,000
**Rat**	Alexa Fluor	Goat	1:10,000

Table S2. Univariate and multivariate regression analyses with all three spinal cord subregions included in analyses. All cell counts are measured at the level of the lesion, without pooling of cell counts from either of the spinal cord subregions. On the left are shown mean (± SE) counts for the immune and neuroglial progenitor markers. P values are estimated for individual comparisons using bootstrap analysis. Bolded are regressors with $p < 0.05$. On the right are shown counts of markers co-stained with BrdU. At the bottom of the table are shown the results of multivariate stepwise regression analysis, which yielded a significant model associated with stimulation for the regressors on the left side of the table, but not for those on the right side of the table. Abbreviations: Stim., stimulation; NS, not significant.

**Immune/Precursor Cells**				**Proliferation**			
**Marker**	**Control**	**Stim.**	**P**	**Markers**	**Control**	**Stim.**	**P**
				BrdU	587 ± 50	519 ± 49	NS
**CD68**	**300 ± 39**	**224 ± 28**	**0.043**	CD68/BrdU	88 ± 20	71 ± 20	NS
CD68/iNOS	16 ± 7	30 ± 10	NS				
CD68/Arg-1	21 ± 6	36 ± 11	NS				
GFAP	187 ± 29	185 ± 29	NS	GFAP/BrdU	55 ± 6	67 ± 10	NS
NG2	181 ± 41	252 ± 64	NS	NG2/BrdU	49 ± 13	59 ± 17	NS
**Sox2**	**384 ± 61**	**250 ± 42**	**0.016**	Sox2/BrdU	74 ± 11	64 ± 4	NS
**Blbp**	**181 ± 38**	**349 ± 67**	**0.000**	**Blbp/BrdU**	**14 ± 2**	**22 ± 6**	**0.047**
Stepwise regression model: NRM LFS ≈ Blbp + CD68 ($F_2 = 51.3$, $p < 0.001$)				Stepwise regression model: N/A			
